# A unique subpopulation of wild-type neurons recapitulating familial Alzheimer’s disease phenotypes

**DOI:** 10.1038/s41419-025-07934-0

**Published:** 2025-08-09

**Authors:** Midori Yokomizo, Michael Sadek, Emily Williams, Mei C. Q. Houser, Natalia Wieckiewicz, Sebastian Torres, Oksana Berezovska, Masato Maesako

**Affiliations:** MassGeneral Institute for Neurodegenerative Disease, Massachusetts General Hospital, Harvard Medical School, Charlestown, MA USA

**Keywords:** Alzheimer's disease, Mechanisms of disease

## Abstract

Mutations in the genes encoding APP, Presenilin-1 (PSEN1), and PSEN2 result in early-onset Alzheimer’s disease (AD). Previous studies, using iPSC-derived neurons and/or knock-in mice, elucidated the characteristics of neurons expressing familial AD (fAD) mutations. Here, we employ biochemical and state-of-the-art fluorescence imaging assays and report the discovery of a unique subpopulation of wild-type neurons strikingly recapitulating key phenotypes previously identified in the fAD neurons, including the favored production of longer over shorter β-amyloid (Aβ) peptides, endo-lysosomal abnormalities, and increased vulnerability phenotypes in response to toxic insults. Importantly, mechanistic studies define inefficient γ-secretase and impaired endo-lysosomes as the upstream events of increased neuronal susceptibility. This discovery of the unique population of neurons with disease phenotypes would open a new avenue to develop novel therapeutics targeting neuronal vulnerability.

## Introduction

Alzheimer’s disease (AD) is the most common cause of dementia. Although the AD-related death rate has risen, there is no sufficiently effective treatment to cure the fatal disease. The deterministic mutations causing early-onset familial AD (fAD) have been identified in the genes encoding APP, Presenilin 1 (PSEN1), or PSEN2, which account for 1 to 5% of all cases. PSEN genes harbor over 300 mutations and represent ~90% of all mutations associated with fAD (https://www.alzforum.org/mutations). In contrast, most cases manifest late-onset sporadic AD (sAD) in which complex genetic, epigenetic, age-related, and environmental factors are thought to dominate. Although the age of onset is significantly different and there are slightly distinct clinical presentations, people with sAD and those with fAD display overall similar clinical and pathological features and trajectories [1, 2]. Nevertheless, whether these two genetically distinct forms share similar causative pathways or have fundamentally different instigating factors that lead to similar clinicopathology is unclear.

APP is one of the substrates of γ-secretase in which PSEN serves as the catalytic core [3, 4]. The N-terminally truncated APP (C99) is first processed by γ-secretase at the epsilon cleavage site (so-called endopeptidase-like activity of γ-secretase), generating the membrane-embedded 48 or 49 amino acid β-amyloid (Aβ) and corresponding intracellular domain fragments. Then, Aβ48 and 49 are cleaved by γ-secretase in a stepwise manner (known as carboxypeptidase-like processivity), generating various Aβ peptides ranging from 37 to 43 amino acids [5, 6]. fAD-causing PSEN mutations affect, to a certain degree, its endopeptidase-like activity and significantly impair the γ-secretase’s carboxypeptidase-like processivity, resulting in the predominant production of longer Aβ peptides (e.g., Aβ42) [7–9]. Aβ42 is highly prone to aggregation [10, 11] and forms the core of senile plaques [12], one of the major pathological hallmarks of AD. In addition, findings in patients’ iPSC-derived neurons have suggested that the endo-lysosomal compartments are dysregulated in neurons encoding fAD mutations [13, 14]. Furthermore, studies employing knock-in mouse models have demonstrated that neurons expressing fAD-causing PSEN display increased vulnerability phenotypes in response to toxic insults and aging [15–18]. Nevertheless, whether and how those molecular and cellular events synergically drive fAD neurodegeneration remains unclear.

Considering AD genetics and neuropathology, the importance of APP processing by γ-secretase is no longer in reasonable doubt. Still, how endogenous γ-secretase activity is *spatiotemporally* regulated and the consequences of changes in γ-secretase activity remains elusive. To fill the gap in knowledge, we have recently developed genetically encoded Förster resonance energy transfer (FRET)-based biosensors, which enable for the first time recording endogenous γ-secretase (i.e., endopeptidase-like) activity, on a cell-by-cell basis, over time, in live/intact neurons [19, 20]. In agreement with previous various studies [e.g., 21–23], our new biosensors verified predominant γ-secretase activity and Aβ generation within the endo-lysosomal compartments in living neurons [24]. The biosensors also suggested limited substrate specificity of γ-secretase in live cells [25], consistent with recent cryo-EM studies of the γ-secretase complex enfolding a fragment of APP or Notch1 [26, 27]. Importantly, our FRET-based biosensors have allowed for the first time to visualize the cell-by-cell heterogeneity in endogenous γ-secretase activity in vitro and in vivo [19, 28].

This study aims to further determine the exact consequences of heterogeneity in endogenous γ-secretase activity in individual neurons. Using a newly developed cell sorting approach, we discovered a unique subpopulation of wild-type neurons displaying not only diminished γ-secretase endopeptidase-like activity but also decreased carboxypeptidase-like function, resulting in increased production of longer Aβ as opposed to shorter Aβ. This unique cell population displays abnormally enlarged endo-lysosomes. Furthermore, we showed that this neuronal population is particularly vulnerable in response to toxic insults such as excitotoxicity and oxidative stress. These findings reveal the existence of a unique subpopulation of wild-type neurons strikingly recapitulating the phenotypes of neurons expressing fAD PSEN mutations. Lastly, our mechanistic studies indicate that inefficient γ-secretase and dysfunctional endo-lysosomes are upstream of increased neuronal vulnerability. Our discovery of the unique neuronal population provides insight into the mechanism that links familial and sporadic cases and opens a new avenue to develop new therapies for AD by stimulating neuronal resilience.

## Material and methods

### Reagents

Propidium Iodide (PI) was purchased from Thermo Fisher Scientific (Waltham, MA). LysoPrime Green was purchased from Dojindo Molecular Technologies, Inc. (Rockville, MD). Chloroquine (CQ), DMSO, 4,4’-dithiodipyridine (DTDP), L-glutamate (Glu), L-Leucyl-L-Leucine methyl ester (LLOMe) were from Sigma-Aldrich (St. Louis, MO), Bafilomycin A1 (Baf A1) was from Cell Signaling Technology, Inc (Dover, MA), and γ-secretase inhibitor: DAPT was from Abcam (Cambridge, UK).

The AAV packaging cDNA of the C99 Y-T biosensor under the human synapsin (hSyn) promoter (stereotype 8: 4.95 × 1013 GC/ml), the C99 720-670 biosensor (stereotype 8: 4.36 ×1013 GC/ml), and the Yellow Cameleon 3.6 (YC3.6) (2.74 × 1012 GC/ml) were generated by the University of Pennsylvania Gene Therapy Program vector core.

### Neuronal culture

The Papain Dissociation System (Worthington Biochemical Corp, Lakewood, NJ) was used to dissociate the cortex primary neurons from CD1 mouse embryos at E14-16 (Charles River Laboratory, Wilmington, MA). The neurons were cultured in Neurobasal medium plus 2% B27 supplement, 1% GlutaMAX, and 1% penicillin-streptomycin (Thermo Fisher Scientific) for 12–15 days in vitro (DIV).

### Confocal microscopy

An Olympus FV3000RS Confocal Laser Scanning Microscope (Tokyo, Japan) was used for fluorescence and FRET imaging. A suitable CO2 concentration and a heating for live-cell imaging was maintained using a CO2/heating unit (Tokai-Hit, Fujinomiya, Japan). The TruFocus Z drift compensation module was used to maintain focus during time-lapse imaging. 10x/0.40NA and 40x/0.95NA objectives were used for image acquisition.

A laser at 488 nm was used for the excitation of LysoPrime Green, and emission was detected within 500–540 nm. A laser at 561 nm was used to excite PI and its emission was detected within 600–680 nm. For FRET, a laser at 405 nm was used to excite mTurquoise-GL in the C99 Y-T biosensor or ECFP in the YC3.6 indicator and the emitted fluorescence was simultaneously detected at 460–480 nm (mTurquoise-GL, ECFP), 520–540 nm (YPet, CpVenus) using the Standard (Multi-alkali photomultiplier tube (PMT)). A laser at 640 nm was used to excite miRFP670 in the C99 720-670 biosensor, and the emitted fluorescence was detected at 660-680 nm (miRFP670), and 700–720 nm (miRFP720) using High-sensitive (Cooled GaAsP PMT) detector. ImageJ was used to measure fluorescent intensity in regions of interest (ROIs), and pseudo-color FRET images were generated in MATLAB (The MathWorks, Natick, MA). The data acquisition and analysis were conducted by independent investigators in a blinded fashion.

### Fluorescence Activated Cell Sorting (FACS)

The SORP 4 Laser BD FACSAria II Cell Sorter (BD Biosciences, Franklin Lakes, NJ) in the MGH Pathology, Flow and Mass Cytometry Core was used to sort neurons with different endogenous γ-secretase activity. Briefly, PI was first used to remove dead neurons, and neurons expressing the C99 Y-T biosensor were excited by a 405 nm laser and the emitted fluorescence was simultaneously detected by 425–475 nm (mTurquoise-GL) and 500–550 nm (YPet) emission channels. The designated 20% of neurons exhibiting the highest YPet/mTurquoise-GL ratios (i.e., 500–550 nm/425–475 nm emission ratios) and the same proportion of neurons displaying the lowest ratios as control were sorted.

### ELISA

Aβ 38 and Aβ 42 were measured using Human Aβ 38 and Aβ 42 ELISA kits, respectively (Immuno-Biological Laboratories, Inc., Minneapolis, MN).

### Western blotting

Protein concentration was measured using the BCA Protein Assay kit (Thermo Fisher Scientific). After mixing the samples with NuPAGE^TM^ LDS Sample Buffer and Sample Reducing Agent (Thermo Fisher Scientific), the samples were boiled, subjected to SDS-PAGE on NuPAGE^TM^ 4–12% Bis-Tris Protein gels (Thermo Fisher Scientific), and transferred to nitrocellulose membranes (Thermo Fisher Scientific) using the BioRad Wet electroblotting system (BioRad, Hercules, CA). The membranes were then incubated with primary and corresponding fluorophore-conjugated secondary antibodies, followed by the development using the LI-COR Odyssey CLx scanner digital imaging system (LI-COR Biosciences, Lincoln, NE).

### Statistical analysis

GraphPad Prism 9 (GraphPad Software, La Jolla, CA) was used for statistical analysis. The D’Agostino and Pearson omnibus normality test was used to examine the Gaussian distribution of the data. Unpaired *t*-tests, one-sample *t*-tests, Mann–Whitney *U* tests, one-way, and repeated ANOVAs were used to compare the data. The Pearson correlation coefficient was measured for correlation analysis. The sample size calculation was based on previous publication and power calculations [19, 24]. At least three independent experiments were performed to ensure the reproducibility of the results. In graphs, the mean and standard deviation (SD) were used as the center value and error bar, respectively.

## Results

### A subpopulation of wild-type neurons favors longer over short Aβ generation

**Fig. 1:**
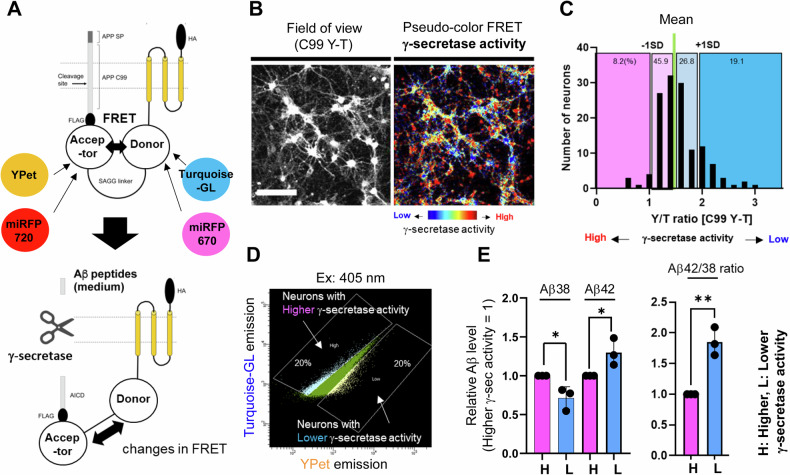
Impaired carboxypeptidase-like trimming activity of γ-secretase in a selected population of neurons. **A** A schematic presentation of the C99 YPet-mTurquoise-GL (C99 Y-T) and its near-infrared analog, the C99 miRFP720-miRFP670 (C99 720-670) biosensors for recording endogenous γ-secretase activity. **B** Endogenous γ-secretase activity is recorded and visualized using the C99 Y-T FRET biosensor. Scale bar 100 μm. **C** Quantification and histogram show that endogenous γ-secretase activity is heterogeneously regulated on a cell-by-cell basis. **D** 20% of neurons displaying the highest and lowest γ-secretase activity are sorted out using FACS, and (**E**) their cell lysates were subjected to human Aβ 38 and 42 ELISA. H high, L low γ-secretase activity. *N* = 3 independent experiments, one-sample *t*-test, *p < 0.05.

Our recent development of FRET-based biosensors [19, 20] and microscopy-based fluorescence imaging have allowed us to detect heterogeneously regulated endogenous γ-secretase activity, on a cell-by-cell basis, in live neurons in vitro [19] and in vivo [28]. Here, we aim to further determine the consequences of changes in endogenous γ-secretase activity in individual neurons. As such, we cultured primary neurons from the cortex of CD1 wild-type mouse embryos, expressed the C99 YPet-mTurquoise-GL (C99 Y-T) biosensor containing human APP C99 [19], and recorded endogenous γ-secretase activity on a cell-by-cell basis (Fig. 1A, B). The cleavage of APP C99 within the C99 Y-T biosensor by γ-secretase triggers the production and release of Aβ peptides and APP intracellular domain Y-T (AICD Y-T). Furthermore, this cleavage results in a change in the proximity and/or orientation between mTurquoise-GL (donor) and YPet (acceptor), decreasing FRET between the donor and acceptor fluorophores, which can be recorded as a decreased Y/T ratio in primary cortical neurons [19] (Fig. 1A). We uncovered that approximately 20% of neurons exhibited Y/T FRET ratios being over Mean + 1 SD (Fig. 1B, C), indicating significantly diminished γ-secretase activity in this subpopulation of neurons. Since fAD-causing PSEN mutations are known to reduce γ-secretase proteolytic efficiency [7–9], we decided to further characterize this unique 20% cell population. To determine whether the neurons with diminished γ-secretase endopeptidase-like activity also exhibit impaired γ-secretase processivity, we employed a fluorescence-activated cell sorter (FACS) in combination with FRET to physically separate and extract the neuronal population with diminished γ-secretase activity (Fig. 1D). In this experiment, the C99 Y-T biosensor was excited by a 405 nm laser, and the fluorescence emission from the donor and acceptor was simultaneously detected by 425–475 nm and 500–550 nm emission channels, respectively. Then, the designated 20% of neurons exhibiting the highest Y/T ratios (i.e., low endopeptidase activity) and the same proportion of neurons displaying the lowest ratios as control were sorted. Lastly, the physically separated neurons were lysed in RIPA buffer and subjected to Human Aβ ELISA. Interestingly, we found that Aβ 38 levels were significantly decreased in the neurons with diminished γ-secretase activity (i.e., high Y/T ratios). In contrast, Aβ 42 levels were significantly increased (Fig. 1E left), resulting in significantly higher Aβ 42/38 ratios in the neurons with diminished γ-secretase activity (Fig. 1E right). These results indicate that in addition to endopeptidase activity, the carboxypeptidase-like processivity of γ-secretase is also down-regulated, further evidencing the inefficiency of γ-secretase in this unique subpopulation of neurons.

### Enlarged endosomes and lysosomes in the wild-type neurons with inefficient γ-secretase

**Fig. 2:**
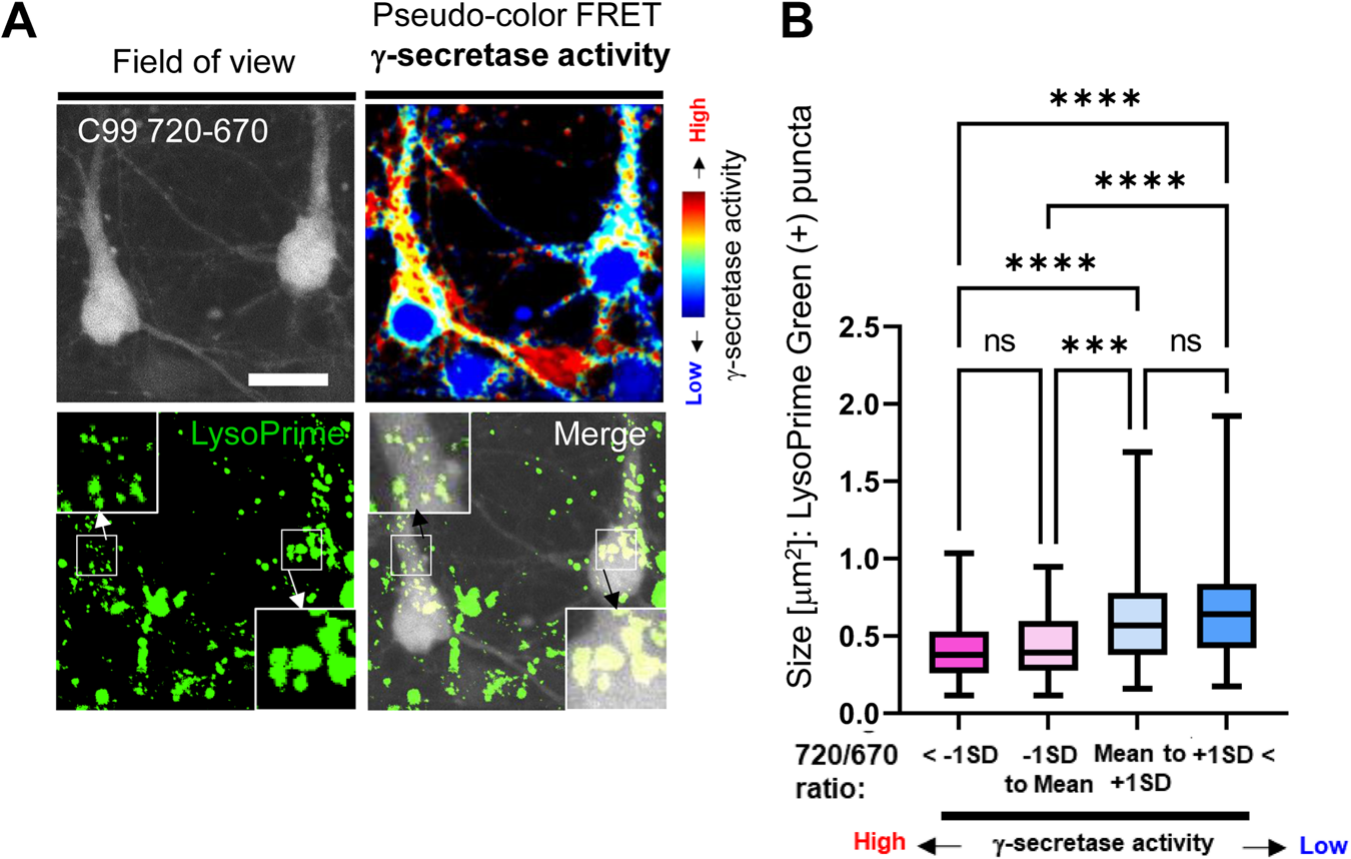
Enlarged endo-lysosomes in the neurons with inefficient endogenous γ-secretase. **A** Endosomes and lysosomes in the neurons expressing the C99 720-670 biosensor were labeled by LysoPrime Green. The 720/670 FRET ratio and LysoPrime Green fluorescence were used to determine γ-secretase activity and the size of endo-lysosomes on a cell-by-cell basis, respectively. Scale bar 20 μm. **B** Neurons were divided into four groups based on γ-secretase activity (720/670 ratio: below -1 standard deviation (SD), −1SD to Mean, Mean to +1 SD, and over +1 SD), and the size of endo-lysosomes were compared among the groups. *N* = 58–129 neurons. One-way ANOVA, n.s. not significant, ***<0.001, ****<0.0001.

Since the γ-secretase with fAD mutant PSEN is inefficient, our discovery of the unique subpopulation of wild-type neurons exhibiting inefficient endogenous γ-secretase led us to hypothesize that this cell population also recapitulates other fAD characteristics. Studies characterizing patient iPSCs-derived neurons suggest that fAD mutations cause the swelling of endosomes and lysosomes [13, 14]. Therefore, we next examined if wild-type neurons with insufficient γ-secretase exhibit enlarged endo-lysosomes. As such, we expressed the near-infrared C99 720-670 biosensor [20] in primary neurons, followed by incubation with LysoPrime Green, a pH-resistant, endo-lysosomes-labeling green fluorescent dye (Fig. 2A). First, cellular γ-secretase activity was measured by detecting the FRET signal from the C99 720-670 biosensor in individual neurons. Then, we assigned the analyzed neurons into four groups with different γ-secretase activities based on the Mean of the 720/670 FRET ratio and ± 1 SD. Lastly, we measured the size of LysoPrime Green positive puncta in individual neurons, which was then compared among the groups (Fig. 2B). Of note, the C99 720-670 biosensor contains miRFP670 and miRFP720 as the donor and acceptor, spectrally compatible with visible-range fluorophores [20, 24, 25]. We found the size of endosomes and lysosomes in the neurons with 720/670 FRET ratios being over the Mean + 1 SD (i.e., those exhibiting lower γ-secretase activity) was significantly larger than that in neurons with the FRET ratio below the average Mean (Fig. 2B). Notably, the size of cell body is not different among the neurons with different γ-secretase activity (Fig. 2-Supplement). These results suggest that the neurons with inefficient γ-secretase exhibit enlarged endo-lysosomes compared to those with active γ-secretase.

### Increased vulnerability phenotypes in the selective neuronal population with inefficient γ-secretase

**Fig. 3:**
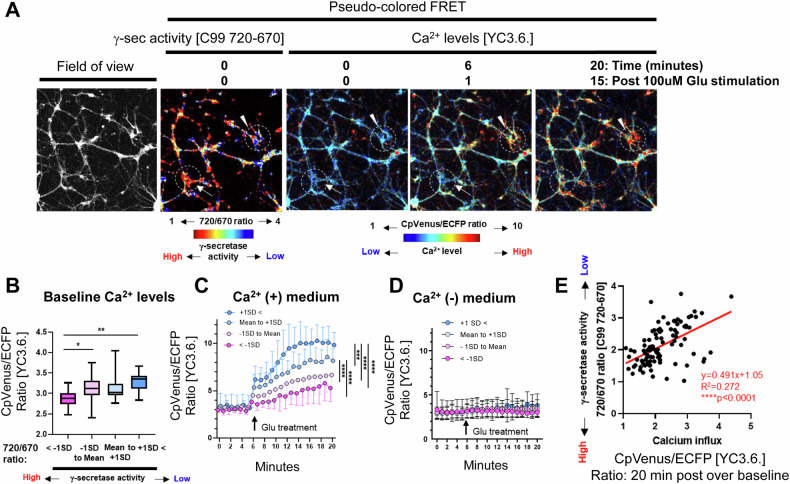
Ca^2+^ dysregulation in the neurons with inefficient endogenous γ-secretase. **A** Baseline γ-secretase activity and calcium levels before and after 100 μM Glu stimulation were measured using the C99 720-670 and YC3.6 sensors in individual neurons, respectively. **B** The neurons were divided into four groups based on γ-secretase activity (720/670 ratio: below -1 standard deviation (SD), −1SD to Mean, Mean to +1 SD, and over +1 SD), and baseline Ca^2+^ levels were compared among the groups. *N* = 11–49 neurons. One-way ANOVA, *<0.05, **<0.01 (**C**) Post 5 min recording baseline Ca^2+^ levels, the neurons were treated with 100 μM Glu. Then, the changes in Ca^2+^ levels were recorded overtime for 15 min and compared among the four groups with different γ-secretase activity. Repeated ANOVA, ****<0.0001. **D** In Ca^2+^-free medium, Glu-induced Ca^2+^ influx was not detectable. *N* = 16–57 neurons. **E** The baseline 720/670 ratio, representing γ-secretase activity, correlates with the magnitude of Ca^2+^ influx (YC3.6. FRET ratio 20 min post-Glu stimulation was divided by that of before stimulation). *N* = 99 neurons, Y = 0.491X + 1.05, R^2^ = 0.272, Pearson correlation coefficient, ****<0.0001.

Earlier studies in fAD PSEN1 knock-in mice also uncovered that neurons with fAD mutations exhibit increased vulnerability phenotypes such as Ca^2+^ overload and impaired membrane integrity in response to toxic insults (e.g., Glutamate (Glu)-induced excitotoxicity) [15, 16]. Therefore, we examined if the wild-type neurons with inefficient γ-secretase are more vulnerable to toxic insults than those with normal γ-secretase. As such, we first monitored γ-secretase activity and cytoplasmic Ca^2+^ levels in individual neurons by employing live-cell multiplexed FRET imaging. In this experiment, we co-expressed the C99 720-670 [20] and the YC3.6. Ca^2+^ reporter probes [29] in primary neurons. Baseline γ-secretase activity was first reported on a cell-by-cell basis using the C99 720-670 biosensor. Then, cytoplasmic Ca^2+^ levels were measured before (5 min) and after (15 min) exposure to 100 μM Glu by recording the FRET signal from the YC3.6. probe (Fig. 3A). We found that the baseline Ca^2+^ level was significantly increased in the neurons with inefficient γ-secretase (Fig. 3A, B). Furthermore, the peak and sustained components of the Glu-induced Ca^2+^ overload were more significant in the neurons with inefficient γ-secretase than those with active γ-secretase (Fig. 3A, C). Notably, Ca^2+^ levels did not significantly increase in the absence of Ca^2+^ in the culturing medium (Fig. 3D), suggesting extracellular Ca^2+^ as the main source of Ca^2+^ overload detected in the neurons. Lastly, we calculated the ratio of CpVenus/ECFP (i.e., acceptor over donor fluorophore of the YC3.6. Ca^2+^ reporter probe) at time 20 min over that at t = 0, which is indicative of a relative increase in Ca^2+^ levels post-Glu exposure. The correlation of the CpVenus/ECFP ratio with baseline γ-secretase activity was analyzed on a cell-by-cell basis. We found a significant positive correlation between the CpVenus/ECFP t = 20 min over t = baseline and the 720/670 ratios (Fig. 3E), suggesting that neurons with lower γ-secretase activity exhibit a more significant increase in cytoplasmic Ca^2+^ levels in response to Glu treatment.

**Fig. 4:**
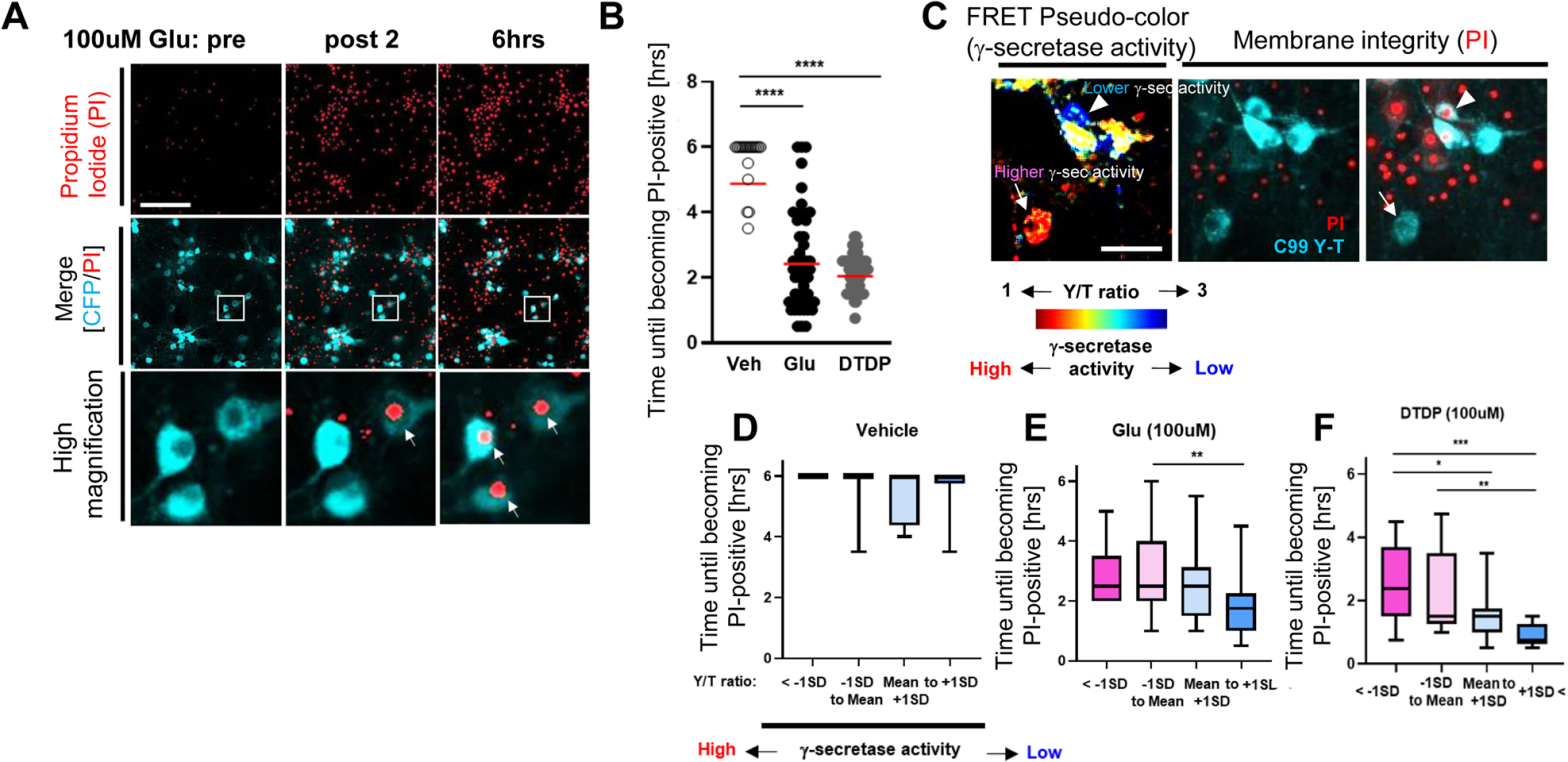
Increased membrane permeability in response to toxic insults in the neurons with inefficient endogenous γ-secretase. **A** Neurons expressing the C99 Y-T FRET biosensor were incubated with 100 μM Glu, 100 μM DTDP, or vehicle in the presence of 5 μg/mL propidium iodide (PI), and the time until becoming PI positive was measured on a cell-by-cell basis. Scale bar 100 μm. **B** Both Glu and DTDP significantly decreased the time until PI (+) compared to vehicle, but Individual neurons differently became PI positive. *N* = 49–57 neurons. One-way ANOVA, ****<0.0001. **C** Baseline γ-secretase activity was measured using the C99 720-670 biosensor and the neurons were divided into four groups based on γ-secretase activity (720/670 ratio: below -1 standard deviation (SD), −1SD to Mean, Mean to +1 SD, and over +1 SD), and the time until becoming PI (+) after treatment with vehicle (*N* = 6–31 neurons) (**D**), 100 μM Glu (*N* = 33–34 neurons) (**E**), or 100 μM DTDP (*N* = 23 neurons) (**F**) was compared among the groups. Scale bar 30 μm. One-way ANOVA, *<0.05, **<0.01, ***<0.001.

To further verify the increased vulnerability to toxic insults in the neurons with inefficient γ-secretase, we employed time-lapse imaging to quantitatively assess membrane permeabilization in individual neurons. In this new assay, we expressed the C99 Y-T biosensor in primary neurons, followed by treatment with insults such as Glu (100 μM), DTDP oxidant (100 μM), or vehicle control in the presence of propidium iodide (PI). PI is a cell impermeable dye that releases fluorescence upon binding to DNA if the cell membrane is compromised and thus can specifically label dead and dying neurons (Fig. 4A). As expected, Glu or DTDP treatment significantly shortened the time until neurons became PI-positive compared to vehicle treatment (Fig. 4B), verifying the reliability of Glu/DTDP-induced toxicity and labeling of the dead neurons by PI. We found that the neurons with inefficient γ-secretase displayed significantly shorter time until becoming PI (+) compared to those with higher activity in response to Glu (Fig. 4C, E) or DTDP (Fig. 4F). These results suggest that the neurons with inefficient γ-secretase are more vulnerable to excitotoxicity and oxidative stress, and changes in endogenous γ-secretase activity could be mechanistically linked to neuronal vulnerability.

### Inefficient γ-secretase and dysfunctional endo-lysosomes are the upstream events of increased neuronal vulnerability

**Fig. 5:**
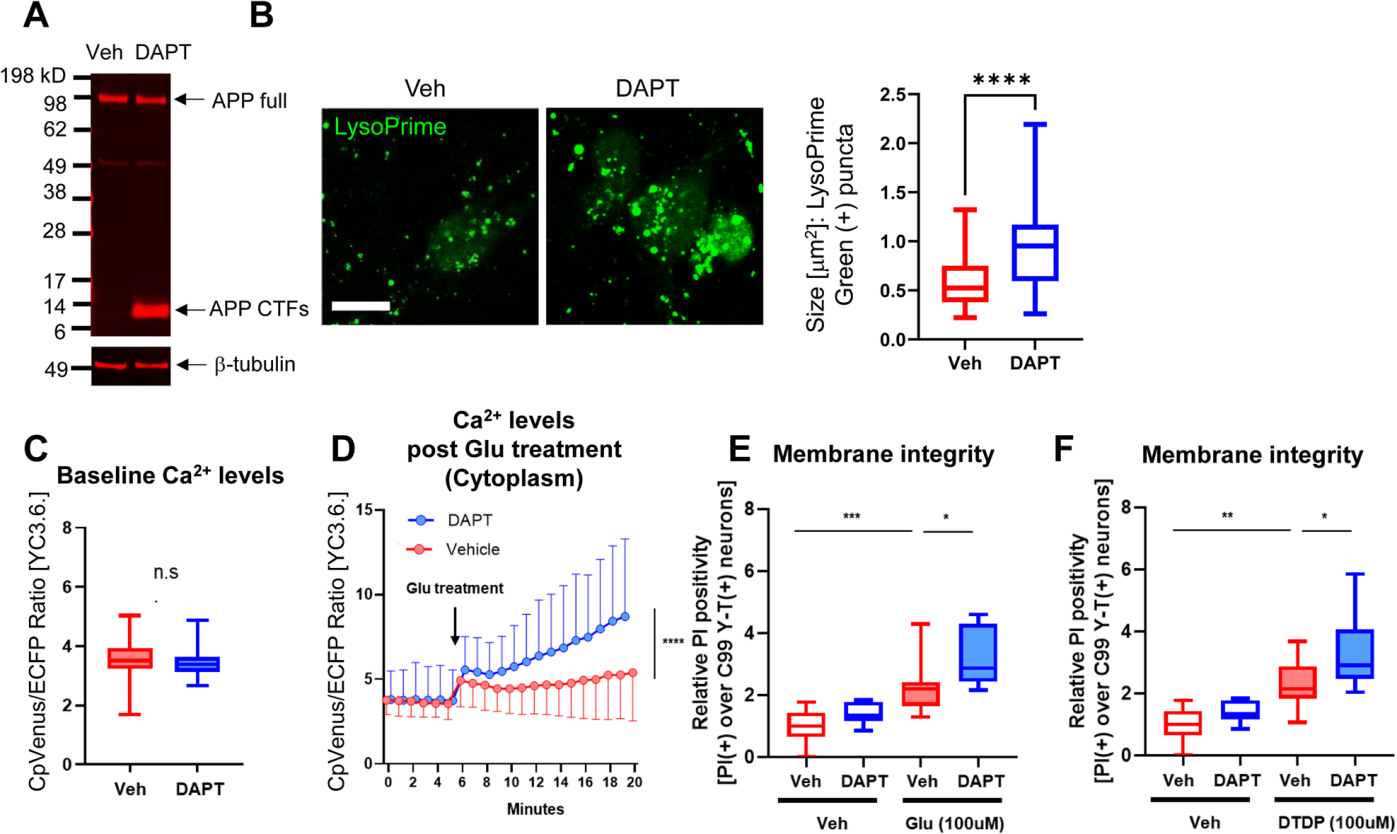
Enlarged endo-lysosomes and increased vulnerability phenotypes in the neurons treated with γ-secretase inhibitor. **A** Neurons were treated with 1 μM DAPT or vehicle control for 16 h. The accumulation of APP-C-terminal fragments (APP-CTFs) is indicative of γ-secretase inhibition. **B** The size of endo-lysosomes in the neurons treated with 1 μM DAPT or vehicle control was assessed by LysoPrime Green. Scale bar 20 μm. *N* = 93–97 neurons. Mann–Whitney *U* test, ****<0.0001 (**C**) Neurons expressing the YC 3.6. sensor were treated with 1 μM DAPT or vehicle control for 16 h, and baseline Ca^2+^ levels were compared. *N* = 59–80 neurons. n.s.: not significant (**D**) Post 5 min recording baseline Ca^2+^ levels, the neurons pre-incubated with DAPT or vehicle were treated with 100 μM Glu, and the changes in Ca^2+^ levels were recorded overtime for 15 min. *N* = 61–81 neurons. Repeated ANOVA, ****<0.0001. **E** The neurons expressing the C99 Y-T biosensor were pre-incubated with 1 μM DAPT or vehicle control for 16 h, followed by 100 μM Glu, (**F**) or 100 μM DTDP for 30 min. Relative numbers of PI (+) neurons over those expressing the C99 Y-T biosensor were measured from 11–13 independent experiments (Veh-veh set as 1). One-way ANOVA, *<0.05, **<0.01, ***<0.001.

We uncovered that the neurons with inefficient endogenous γ-secretase display enlarged endosomes and lysosomes, and increased vulnerability phenotypes such as Ca^2+^ overload and impaired membrane integrity; however, the cause-and-effect relationship is unestablished. Therefore, we first examined whether pharmacological inhibition of γ-secretase recapitulates the phenotypes uncovered in the wild-type neurons with inefficient γ-secretase. As such, primary neurons were incubated with 1 μM DAPT, a potent γ-secretase inhibitor [30], or vehicle control for 16 h. Effective inhibition of γ-secretase was verified by detecting significantly accumulated APP-CTFs, immediate substrates of γ-secretase (Fig. 5A, Fig. 5-Supplement). DAPT-treated neurons were then incubated with LysoPrime Green to measure the size of endosomes and lysosomes. We found significantly larger LysoPrime Green positive puncta in the neurons treated with DAPT than in those with vehicle control (Fig. 5B), which is consistent with previous literature that employed immunocytochemistry-based approaches [13, 14].

Next, we examined if pharmacological inhibition of γ-secretase results in Ca^2+^ overload and impaired membrane integrity in response to toxic insults. For this, primary neurons expressing the YC3.6. Ca^2+^ reporter were pre-treated with either 1 μM DAPT or vehicle for 16 h, followed by treatment with 100 μM Glu. Then, cytoplasmic Ca^2+^ levels were measured before (5 min) and after (15 min) exposure to Glu. We found that although the baseline Ca^2+^ levels were comparable between neurons incubated with DAPT and those with vehicle control (Fig. 5C), Glu treatment significantly increased cytoplasmic Ca^2+^ levels in neurons treated with DAPT compared to those with vehicle control (Fig. 5D). Next, primary neurons expressing the C99 Y-T biosensor were pre-incubated with either 1 μM DAPT or vehicle for 16 h, followed by the treatment with 100 μM Glu, 100 μM DTDP, or vehicle control for 30 min in the presence of PI, and then, the relative number of PI (+) neurons was recorded. We found that DAPT treatment significantly increased the number of PI (+) neurons compared to vehicle control in the neurons exposed to Glu (Fig. 5E) or DTDP (Fig. 5F), indicating that pharmacological inhibition of γ-secretase results in Ca^2+^ overload and impaired membrane integrity in response to toxic insults. Altogether, these results strongly suggest that inefficient γ-secretase is an upstream event of the endo-lysosomal abnormality and increased neuronal vulnerability.

**Fig. 6:**
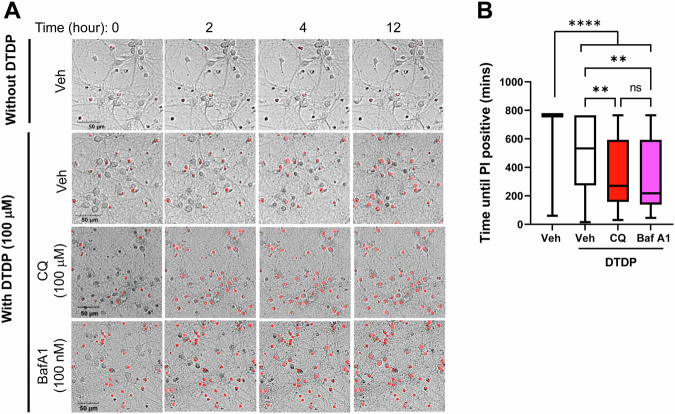
Endo-lysosomes disturbance is the upstream of increased vulnerability. **A** Neurons were treated with 100 μM CQ, 100 nM Baf A1, or vehicle control for 5 h, followed by time-lapse imaging to measure the time until becoming PI (+) on a cell-by-cell basis in response to DTDP treatment. **B** Neurons treated with CQ or Baf A1 exhibited significantly shorter time until becoming PI positive compared to vehicle control. Scale bar 50 μm. *N* = 60–92 neurons. One-way ANOVA, **<0.01, ****<0.0001.

Lastly, we examined if endo-lysosomal dysfunction causes increased neuronal vulnerability. To address this, primary neurons were pre-incubated with 100 μM Chloroquine (CQ), 100 nM Bafilomycin A1 (Baf A1) or vehicle control for 5 h, followed by time-lapse imaging to monitor the time until becoming PI (+) in response to DTDP treatment. We found that although the pre-incubation per se did not significantly increase PI-positive neurons, the neurons treated with CQ, or Baf A1 became PI positive significantly faster (Fig. 6A, B). These results strongly suggest that endo-lysosomal disturbance leads to increased neuronal vulnerability and thus the dysregulated endo-lysosomal system is the upstream event of neuronal vulnerability. Of note, we pre-incubated neurons with 1–10 mM range LLOMe either for 5 or 24 h and found that LLOMe significantly decreased the time until becoming PI-positive in several experiments; however, there was no difference in some experiments (Fig. 6-Supplement).

## Discussion

Our recent development of novel FRET-based biosensors has enabled the detection of heterogeneously regulated endogenous γ-secretase activity on a cell-by-cell basis in live neurons [19, 20, 28]. In this study, we show that the neurons with lower γ-secretase activity favor longer (i.e., Aβ 42) over shorter Aβ (i.e., Aβ 38) generation (Fig. 1), suggesting that carboxypeptidase-like trimming capability of γ-secretase is also down-regulated in the unique cell population. Moreover, this selective neuronal population with inefficient γ-secretase displays enlarged endo-lysosomes (Fig. 2) and increased vulnerability phenotypes, including dysregulated Ca^2+^ homeostasis and impaired membrane integrity (Figs. 3 and 4). Furthermore, our mechanistic studies suggest that inefficient γ-secretase and endo-lysosome abnormalities are the upstream events of increased neuronal vulnerability (Figs. 5 and 6). Given that decreased carboxypeptidase-like activity of γ-secretase [7–9], swelled endo-lysosomes [13, 14] and increased vulnerability [15, 16] were uncovered in the neurons expressing fAD PSEN mutations, our findings suggest that there is a unique subpopulation of wild-type neurons recapitulating these fAD neuronal phenotypes.

Alzheimer’s disease can be either sporadic or caused by inherited autosomal dominant mutations. Since the discoveries three decades ago [31–33], genetic mutations have been so far identified only in the three genes: APP, PSEN1, and PSEN2, with PSEN mutations accounting for most familial cases (https://www.alzforum.org/mutations). Given the facts that PSEN is the catalytic component of γ-secretase [4, 34] and APP is one of the substrates of γ-secretase [3], it has been extensively investigated how FAD PSEN mutations impact APP processing by γ-secretase. Previous studies elucidated that the trimming of longer Aβ to shorter peptides is impaired by the mutations [7–9]. Our study adds an interesting discovery that there is a subpopulation of wild-type neurons that favor producing longer over shorter Aβ (Fig. 1). γ-Secretase is embedded in the lipid membrane bilayer and it is suggested that alteration of membrane properties, thickness in particular, impacts the generation of long vs. short Aβ species by γ-secretase [35–37]. Given that membrane permeability is increased in this newly discovered subpopulation of wild-type neurons (Fig. 4), we assume their membrane properties might be differently regulated.

While one of the significant aspects of this study is the discovery of a unique fAD-like neuronal population that exhibits inefficient γ-secretase (Fig. 1), abnormal endo-lysosomes (Fig. 2), and increased vulnerability phenotypes (Figs. 3 and 4), we also present mechanistic studies to determine their cause-and-effect relationships. First, to establish the causal relationship between inefficient γ-secretase and endo-lysosomal abnormalities, we pharmacologically inhibited endogenous γ-secretase. In agreement with previous studies [13, 14, 38], we found that γ-secretase inhibitor causes endo-lysosome enlargement (Fig. 5), suggesting that inefficient γ-secretase is the upstream event of endo-lysosomal abnormalities. Previously, we have also found alkalized endo-lysosomes in wild-type neurons with inefficient γ-secretase [24]. As potential mechanisms, recent studies proposed that C99/APP-CTFs [14, 39, 40] or even the YENPTY motif in the cytoplasmic region of APP CTFs [38] links inefficient γ-secretase with endo-lysosomal abnormalities (e.g., swelling, pH alterations, Ca^2+^ dysregulation).

Next, we aimed to address whether increased neuronal vulnerability to toxic insults is the consequence of inefficient γ-secretase and endo-lysosome abnormalities. We uncovered that γ-secretase inhibitor results in Ca^2+^ overload and impaired membrane integrity (Fig. 5), suggesting that increased vulnerability is downstream of inefficient γ-secretase. Furthermore, we uncovered that pre-incubation with CQ or Baf A1 leads neurons to be more vulnerable in response to oxidative stress (Fig. 6). This suggests that increased vulnerability is also downstream of endo-lysosomal abnormalities. Together, it is highly presumable that inefficient γ-secretase leads to a dysfunctional endo-lysosome system, which increases neuronal vulnerability. While CQ and Baf A1 consistently decreased the time until becoming PI positive post oxidative stress induction in all three independent experiments that we performed (Fig. 6), we uncovered a different result with a lysosomotropic detergent LLOMe (Fig. 6-Supplement). Both CQ and Baf A1 are known to interfere with endo-lysosomal pH and autophagosome-lysosome fusion [41–44]. On the other hand, LLOMe induces endo-lysosomal membranolysis [45]. Thus, we expect a different mechanism of action between CQ/Baf A1 and LLOMe, e.g., modification of autophagy, may provide more detailed insight into the regulatory mechanism of neuronal vulnerability.

Utilizing the C99 720-670 biosensor with near-infrared spectral properties [20] enabled us to perform multiplexing imaging to better understand how γ-secretase is functionally associated with other key biological events (e.g., the morphology of endo-lysosomes, Ca^2+^ influx) (Figs. 2 and 4). The multiplexing FRET approach presented in this study could have important implications for future research that involves experimental manipulations and simultaneous monitoring of two (or more) molecular processes. Moreover, our time-lapse imaging using propidium iodide (PI) enabled quantitative recording of neuronal death on a cell-by-cell basis (Figs. 3 and 6), highlighting heterogeneously regulated vulnerability to toxic insults in individual neurons. This new functional imaging platform can be utilized for drug screening as well as to determine molecular factors regulating neuronal vulnerability and resilience.

In conclusion, this study reports the discovery of a unique neuronal population exhibiting characteristics similar to those of neurons with fAD PSEN mutations, such as longer Aβ production (Fig. 1), endo-lysosomal swelling (Fig. 2), and increased vulnerability phenotypes (Figs. 3 and 4). Our mechanistic studies reveal that inefficient γ-secretase activity and endo-lysosome abnormalities are the causes of increased neuronal vulnerability to toxicities (Figs. 5 and 6). Future research characterizing this unique subpopulation in more detail will help determine the upstream molecular and cellular events driving neuronal susceptibility and the downstream consequences, which can be utilized to establish molecular markers of selectively vulnerable neurons.

## Supplementary information

Figure 2-Supplement

Figure 5-Supplement

Figure 6-Supplement

## Data Availability

All data generated or analyzed during this study are included in this published article and its supplementary information files.
