# Supplementary material for: A unique subpopulation of wild-type neurons recapitulating familial Alzheimer’s disease phenotypes
Source: Cell Death Dis. 2025 Aug 9;16(1):604. doi: 10.1038/s41419-025-07934-0 (PMC12335501; doi:10.1038/s41419-025-07934-0)
Supplement: Supplementary file 2 — Figure 5-Supplement [file 41419_2025_7934_MOESM2_ESM.pdf]

**A unique subpopulation of wild-type neurons recapitulating familial Alzheimer's disease phenotypes**

Midori Yokomizo[1], Michael Sadek[1], Emily Williams[1], Mei C.Q. Houser[1], Natalia Wieckiewicz[1], Sebastian Torres[1], Oksana Berezovska[1] and Masato Maesako[1*]

[1]MassGeneral Institute for Neurodegenerative Disease, Massachusetts General Hospital, Harvard Medical School, 114, 16th Street, Charlestown, MA 02129, USA

[*]Correspondence: Masato Maesako, Ph.D., MMAESAKO@mgh.harvard.edu; Tel.: +1-617-724-2579

# Figure 5 - Supplement

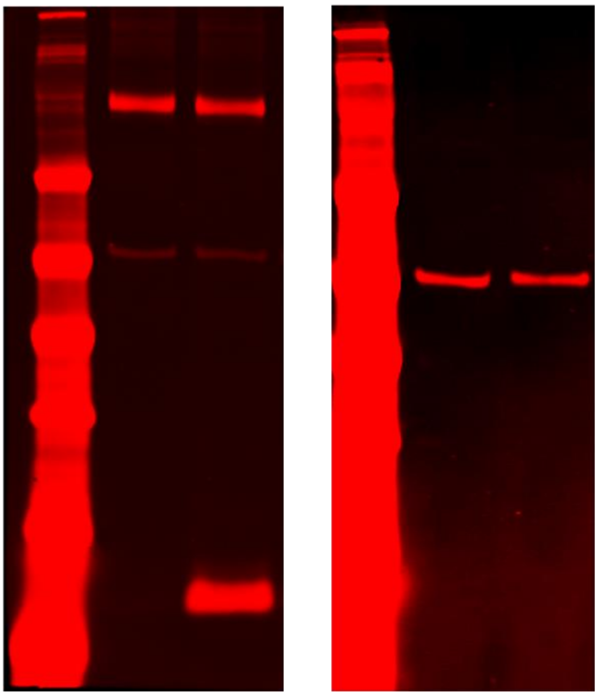

WB: APP C-term WB: beta-tubulin

**The uncropped original western blots of Figure 5A**
