# Supplementary material for: A unique subpopulation of wild-type neurons recapitulating familial Alzheimer’s disease phenotypes
Source: Cell Death Dis. 2025 Aug 9;16(1):604. doi: 10.1038/s41419-025-07934-0 (PMC12335501; doi:10.1038/s41419-025-07934-0)
Supplement: Supplementary file 3 — Figure 6-Supplement [file 41419_2025_7934_MOESM3_ESM.pdf]

A unique subpopulation of wild-type neurons recapitulating familial Alzheimer's disease phenotypes

Midori Yokomizo[1], Michael Sadek[1], Emily Williams[1], Mei C.Q. Houser[1], Natalia Wieckiewicz[1], Sebastian Torres[1], Oksana Berezovska[1] and Masato Maesako[1*]

[1]MassGeneral Institute for Neurodegenerative Disease, Massachusetts General Hospital, Harvard Medical School, 114, 16th Street, Charlestown, MA 02129, USA

[*]Correspondence: Masato Maesako, Ph.D., MMAESAKO@mgh.harvard.edu; Tel.: +1-617-724-2579

Figure 6 - Supplement

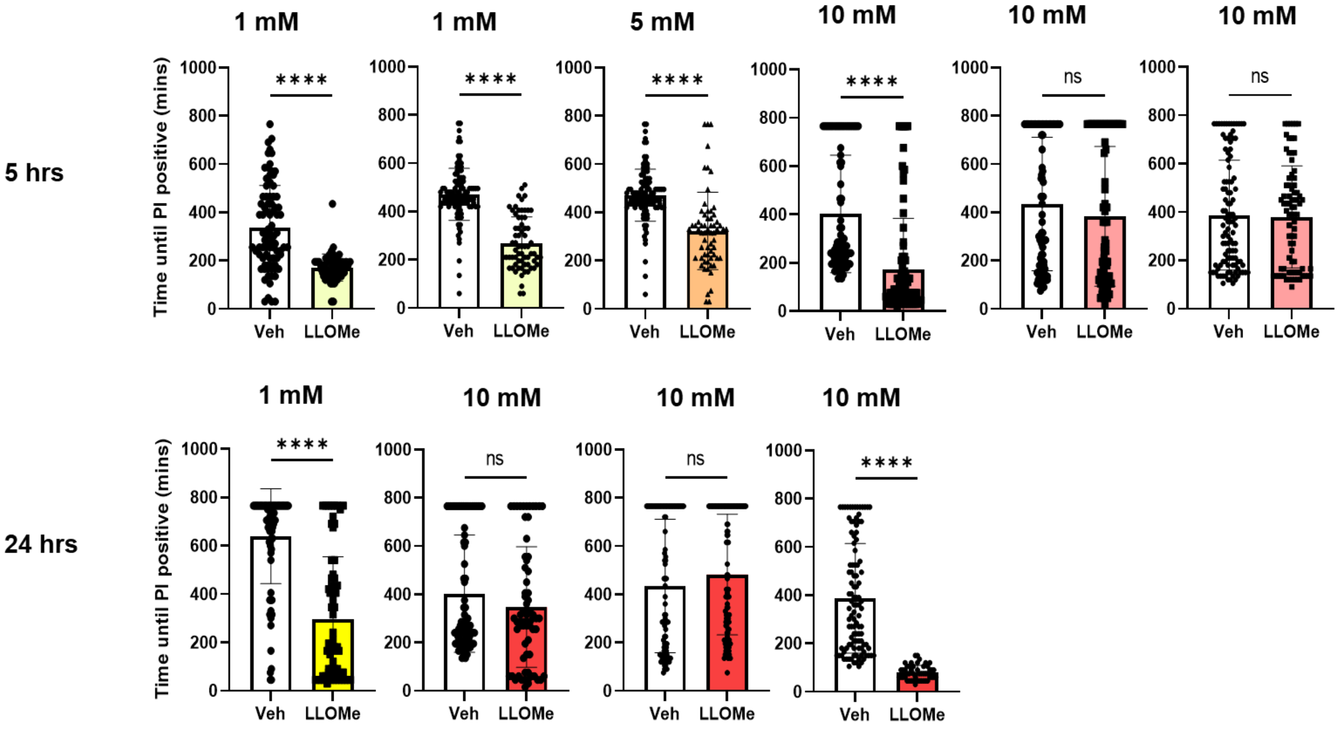

Whereas LLOMe overall decreases the time until becoming PI (+), the effect is more random than CQ and Baf A1: Neurons were treated with 1mM, 5 mM, 10 mM LLOMe or vehicle control for 5 or 24 hours, followed by time-lapse imaging to measure the time until becoming PI (+) on a cell-by-cell basis in response to DTDP treatment. In some experiments, the time until becoming PI (+) is significantly shorter in the neurons treated with LLOMe compared to vehicle control; on the other hand, there is no difference in some experiments. Mann–Whitney U test, n.s. no significance, ****<0.0001.
